# Progress Toward Poliomyelitis Eradication — Afghanistan, January 2022–June 2023

**DOI:** 10.15585/mmwr.mm7238a1

**Published:** 2023-09-22

**Authors:** Adam Bjork, Irfan Elahi Akbar, Sumangala Chaudhury, Mufti Zubair Wadood, Fazal Ather, Jaume Jorba, Maureen Martinez

**Affiliations:** ^1^Global Immunization Division, Global Health Center, CDC; ^2^Polio Eradication Department, World Health Organization, Kabul, Afghanistan; ^3^Polio Eradication Department, World Health Organization, Geneva, Switzerland; ^4^Polio Eradication Department, World Health Organization, Amman, Jordan; ^5^Division of Viral Diseases, National Center for Immunization and Respiratory Diseases, CDC.

**Summary**

**What is already known about this topic?**

Wild poliovirus type 1 (WPV1) remains endemic only in Afghanistan and Pakistan.

**What is added by this report?**

Afghanistan reported two WPV1 cases during 2022 and five during 2023 through June 30. All cases were detected along the Pakistan border, and all patients during 2023 had a history of receipt of ≥16 oral poliovirus vaccine doses. During May 2023, WPV1 circulation was detected for the first time in >2 years in the south region of Afghanistan, where restrictions prohibiting house-to-house vaccination limit the effectiveness of immunization campaigns.

**What are the implications for public health practice?**

Interruption of WPV1 transmission in Afghanistan is attainable and requires regular and unrestricted supplementary immunization activities (mass campaigns), improved surveillance, and strong coordination of vaccination activities with neighboring Pakistan.

## Abstract

When the Global Polio Eradication Initiative began in 1988, wild poliovirus (WPV) transmission was reported in 125 countries. Since 2017, Afghanistan and Pakistan remain the only countries with uninterrupted endemic WPV type 1 (WPV1) transmission. This report describes activities and progress toward polio eradication in Afghanistan during January 2022–June 2023. Two WPV1 cases were reported during January–December 2022 and five during January–June 2023 (as of August 26), all from three provinces in the southeast and east regions bordering Pakistan. All five 2023 patients had reportedly received ≥16 oral poliovirus vaccine doses. WPV1 was detected in sewage samples from a site in the south region in May 2023 and one in the north region in June 2023, the first detections since February 2021 and March 2020, respectively. Restrictions on house-to-house vaccination limit the effectiveness of vaccination campaigns in parts of the south and northeast regions. Because of population movement, the risk for transmission in Afghanistan and Pakistan will remain if WPV1 circulation continues in either country. Despite operational improvements in vaccination activities, interruption of WPV1 transmission in Afghanistan will require committed, uninterrupted efforts, including ongoing coordination with Pakistan on polio eradication activities, to address vaccination coverage gaps that sustain WPV1 circulation.

## Introduction

Worldwide, wild poliovirus (WPV) cases have decreased by more than 99.9% since the Global Polio Eradication Initiative (GPEI) began in 1988, and global eradication of indigenous WPV types 2 and 3 has been certified. However, endemic transmission of indigenous WPV type 1 (WPV1) has never been interrupted in Afghanistan and Pakistan. These countries share a long (1,600 mile [2,600 km]) border frequently traversed by highly mobile populations (*1*). The GPEI 2022–2026 strategic plan set the end of 2023 as the target for interrupting all WPV1 transmission (*2*). Afghanistan reported 56 WPV1 cases during 2020 and four during 2021 (*3*,*4*). After years of active conflict, the Afghanistan government was replaced by Taliban authorities in August 2021. Restrictions on supplementary immunization activities (SIAs)* that prohibited house-to-house oral poliovirus vaccine (OPV) vaccination (the most effective SIA modality) existed for many years in Taliban-held areas. These restrictions remain in parts of the south and northeast regions, where SIA OPV administration is allowed only at health facilities, mosques, and polio vaccination sites.

## Methods

Data and SIA information were provided by the Afghanistan National Emergency Operations Centre, which includes officials from UNICEF, the World Health Organization (WHO), and other GPEI partners. Lot quality assurance sampling (LQAS)^†^ surveys assess district-level SIA quality. Acute flaccid paralysis (AFP) surveillance detects recent onset of weakness among children. Detection of ≥2 nonpolio AFP (NPAFP) cases^§^ per 100,000 children aged <15 years, coupled with collection of adequate stool specimens^¶^ from ≥80% of AFP cases, indicate surveillance that is sufficiently sensitive to detect a case of paralytic polio. Environmental surveillance (ES) for poliovirus in Afghanistan is conducted via systematic sampling of sewage at 37 sites in 17 provinces and virologic testing. Genomic sequencing analyses determine genetic relationships among polioviruses identified in stool and ES specimens. This activity was reviewed by CDC, deemed not research, and was conducted consistent with applicable federal law and CDC policy.**

## Results

### Immunization Activities

WHO and UNICEF estimated national 3-dose OPV coverage in Afghanistan among children aged 12–23 months to be 76% during 2022 and 71% during 2021. In 2015, the Global Commission for the Certification of the Eradication of Poliomyelitis declared wild poliovirus type 2 to be eradicated.^††^ In 2016, Afghanistan and all other OPV-using countries implemented a global synchronized switch from trivalent OPV (containing Sabin-strain types 1, 2, and 3) to bivalent OPV (bOPV, containing Sabin-strain types 1 and 3) and ≥1 dose of inactivated poliovirus vaccine (IPV), which includes all three serotypes. Estimated 1-dose IPV coverage in Afghanistan was 71% in 2022 and 67% in 2021 (*5*). Vaccination coverage among children with NPAFP, on the basis of review of immunization cards and caregiver recall of routine immunization (RI) and SIA vaccination dose histories, serves as a proxy for RI and SIA coverage and allows for subnational level analyses. Among 3,308 infants and children aged 6–59 months with NPAFP in 2022, 67% had a history of receipt of ≥3 RI OPV doses; 17% had never received any RI OPV dose. During 2023 to date, reported ≥3-dose RI OPV coverage improved to 73%, and the percentage of infants and children with no OPV doses received through RI declined to 13%. The percentage of infants and children who never received OPV through RI or SIAs (zero-dose children) decreased from 1.4% in 2022 to 0.8% during 2023. Among Afghanistan’s 34 provinces, 13 (38%) reported zero-dose children with NPAFP during the reporting period; the highest percentages were in provinces in the south region (Kandahar = 6%; Uruzgan = 5%; and Helmand = 4%).

Twelve SIAs were conducted during 2022: six national immunization days (NIDs), three subnational immunization days (SNIDs), and three case-response campaigns. Five SIAs were conducted during January–June 2023: one NID, two SNIDs, and two case-response campaigns. All SIAs used bOPV. The percentage of the target population (children aged <5 years) living in areas where NIDs were conducted without restrictions on house-to-house vaccination varied during 2022 from 50% during January, to 76% during September and was 68% for the March 2023 NID. Reported NID OPV coverage was approximately 100% in areas without restrictions on house-to-house vaccination; in areas with such restrictions, NID coverage ranged from 71% during January 2022 to 86% during March 2023.

LQAS surveys throughout the reporting period included areas with and without restrictions on house-to-house vaccination. One LQAS lot represented a single district, except in some larger urban districts. Total lots assessed per NID increased from 174 during February 2022 to 357 during March 2023. The percentage of lots reported as having passed increased from 51% (March 2022) to 77% (March 2023). Lots in the south and northeast regions constituted 23% of all lots surveyed during the reporting period but 58% of all lots that failed. Only 16% of lots surveyed in the south region passed, and 0% in Kandahar province passed. In the March 2023 NID, 93% of lots passed in districts with no restrictions on house-to-house vaccination, compared with only 6% in districts with restrictions.

Vaccination is offered to children aged ≤10 years along major travel routes throughout Afghanistan and to persons of all ages at two border crossing points with Pakistan. During January 2022–June 2023, a total of 14,106,879 bOPV doses were administered at transit points and 1,690,497 at border crossings.

### AFP Surveillance

Afghanistan’s AFP surveillance network includes 1,932 active surveillance sites that are visited by surveillance officers, 3,251 sites with passive monthly reporting, and 49,870 community-based reporting volunteers. During 2022, the national NPAFP rate was 24 per 100,000 persons aged <15 years (regional range = 16–42); during January–June 2023, the annualized NPAFP rate was 26 (regional range = 18–42) (Table). The percentage of AFP cases with adequate specimens was 94% during both 2022 (regional range = 91%–97%) and 2023 to date (regional range = 89%–98%).

**TABLE:** Acute flaccid paralysis surveillance performance indicators, reported cases of wild poliovirus type 1, and number of environmental specimens with detection of wild poliovirus type 1, by region and period — Afghanistan, January 2022–June 2023*

Region	AFP surveillance performance indicators						No. of WPV1 cases reported			No. of ES samples with WPV1 detected^†^		
	No. of AFP cases		NPAFP rate^§^		% with adequate stool specimens^¶^		2022		2023	2022		2023
	2022	2023	2022	2023**	2022	2023	Jan–Jun	Jul–Dec	Jan–Jun	Jan–Jun	Jul–Dec	Jan–Jun
**All**	**5,368**	**2,876**	**24.3**	**25.5**	**94.4**	**94.2**	**1**	**1**	**5**	**3**	**19**	**32**
Badakhshan	121	59	18.6	17.8	94.2	98.3	0	0	0	0	0	0
Central	981	602	19.5	23.5	97.1	97.0	0	0	0	0	0	0
East	930	479	41.9	41.9	95.1	95.2	0	1	5	3	19	30
North	443	262	16.3	18.8	92.1	90.8	0	0	0	0	0	1
Northeast	496	264	20.1	20.9	93.3	94.3	0	0	0	0	0	0
South	1,060	538	27.8	27.6	91.2	88.5	0	0	0	0	0	1
Southeast	565	264	25.7	23.5	96.3	96.6	1	0	0	0	0	0
West	772	408	26.2	27.1	95.2	96.1	0	0	0	0	0	0

**Abbreviations:** AFP = acute flaccid paralysis; ES = environmental surveillance; NPAFP = nonpolio acute flaccid paralysis; WPV1 = wild poliovirus type 1.

* Data as of July 9, 2023.

^†^ Total number of ES samples by period, January 2022–June 2023.

^§^ Cases per 100,000 persons aged <15 years. The surveillance performance indicator target for countries with poliovirus circulation is three or more NPAFP cases per year per 100,000 persons aged <15 years.

^¶^ Adequate stool specimens are defined as two stool specimens of sufficient quality for laboratory analysis, collected ≥24 hours apart, both within 14 days of paralysis onset, and arriving in good condition at a World Health Organization–accredited laboratory with reverse cold chain maintained, without leakage or desiccation, and with proper documentation.

** Annualized from AFP surveillance data through June 2023.

### Environmental Surveillance

WPV1 was isolated from 22 ES specimens during 2022, all from east region sites (19 from Nangarhar province and three from Kunar province). During January–June 2023, WPV1 was detected in 32 ES samples: 30 from the east (27 from Nangarhar and three from Kunar) and one each from the south (Kandahar province, collected during May) and north (Balkh province, collected during June) regions, the first WPV1 detections in the south and north regions since February 2021 and March 2020, respectively.

### Epidemiology of Poliovirus Cases

**FIGURE 1:**
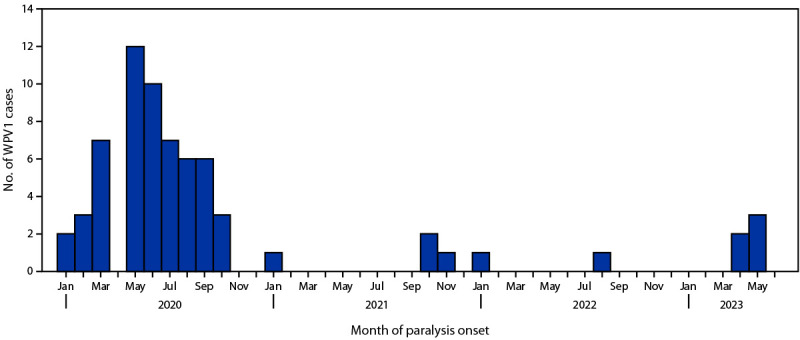
Number of reported cases of polio caused by wild poliovirus type 1 (N = 67), by month of paralysis onset — Afghanistan, January 2020–June 2023* **Abbreviation:** WPV1 = wild poliovirus type 1. * As of August 26, 2023.

**FIGURE 2:**
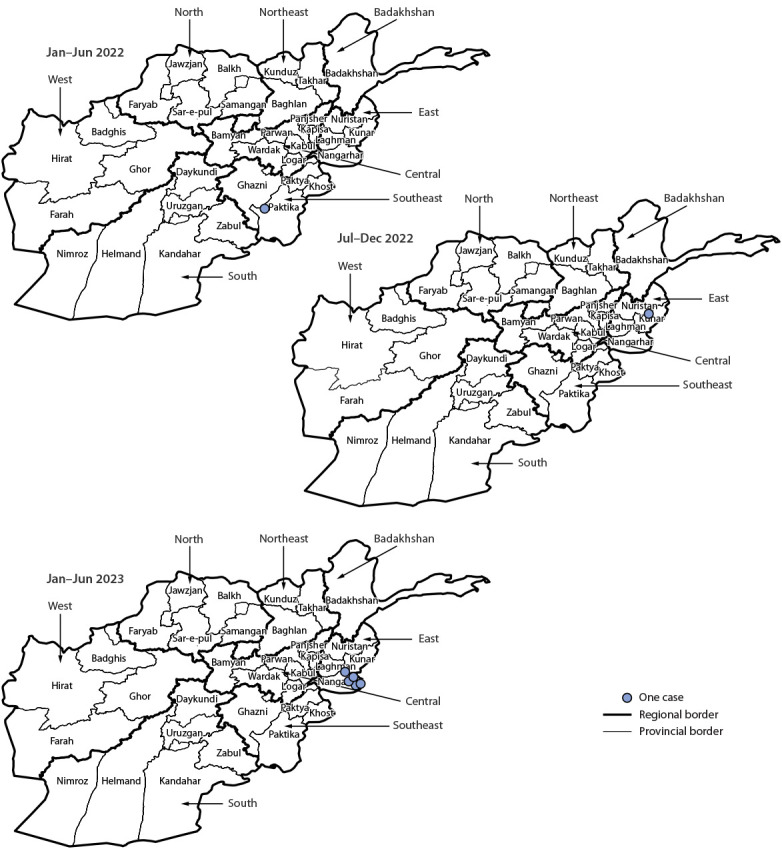
Reported cases of polio caused by wild poliovirus type 1 (N = 7), by region, province, and period — Afghanistan, January 2022–June 2023* * As of August 26, 2023.

After the report of four WPV1 cases during 2021 (one from Ghazni province in the southeast region and three from Kunduz province in the northeast region), two cases were reported during 2022 (one each from Paktika and Kunar provinces in the southeast and east regions, respectively) (Figure 1) (Figure 2). During January–June 2023, five WPV1 cases were reported, all from the eastern province of Nangarhar. The mean patient age at paralysis onset increased from 19 months during 2021 (range = 10–25 months; median = 21 months) to 32 months during 2022 (range = 24–39 months; median = 32 months) and to 66 months during January–June 2023 (range = 30–132 months; median = 48 months). One patient during January 2022–June 2023 (aged 24 months at January 2022 onset) reportedly had never received OPV. The remaining six patients each reportedly received an average of 20 doses via RI and SIA (range = 11–28 doses); all five 2023 patients had received ≥16 total doses each.

### Genomic Sequence Analysis of Poliovirus Isolates

Genomic sequence analysis of the region coding the VP1 capsid protein of poliovirus isolates provided evidence of two genetic clusters (groups of isolates sharing ≥95% of VP1 sequence identity) among recent cases. WPV1 cluster YB3A, the remaining endemic cluster circulating in eastern Afghanistan, was detected in east region AFP cases and environmental specimens from Kunar and Nangarhar provinces during 2022–2023 as well as in environmental specimens from Kandahar (south region) and Balkh (north region) provinces collected during May and June 2023, respectively. A WPV1 cluster YB3C isolate genetically linked to isolates from Pakistan was detected in a January 2022 AFP case in Paktika province near the Pakistan border. Isolates genetically related to circulating YB3A viruses in eastern provinces of Kunar and Nangarhar were detected in ES samples in Khyber Pakhtunkhwa and Punjab provinces, Pakistan (*6*). Two of the five AFP WPV1 viruses detected during 2023 were >1.1% divergent from their closest known genetic matches, suggesting gaps in surveillance, although this level of divergence did not reach the “orphan virus” threshold.^§§^

## Discussion

The geographic distribution of reported polio cases in Afghanistan has narrowed since 2021. The five cases to date during 2023 were from the eastern province of Nangarhar, which conducted the most SIAs during the reporting period. Each of the five patients had reportedly received ≥16 OPV doses through RI and SIAs. The recent scarcity of a reported history of undervaccination among polio patients is consistent with the frequency of SIAs with high reported coverage and with data indicating that malnutrition and diarrheal diseases can interfere with immune response to OPV vaccination; malnutrition affects 54% of Afghanistan’s children (*7*,*8*). The current higher median age at paralysis onset might indicate a shift in WPV1 epidemiology, such that the population most susceptible to infection is now dominated by children above the age of the SIA target age group, who might have been missed in some SIAs when they were younger.

The May 2023 detection of WPV1 in Kandahar, the first detection in the south region since February 2021, indicates the continued need to prioritize this region for future vaccination activities. Southern Afghanistan shares a border with Pakistan and is a historical reservoir for poliovirus transmission, with low OPV coverage and restrictions on house-to-house vaccination.

### Limitations

The findings in this report are subject to at least two limitations. First, outdated target population estimates might have limited the accuracy of reported SIA coverage. These targets were updated during mid-2023 (increasing by 10% nationwide) and are expected to improve the accuracy of coverage estimates for subsequent SIAs. Second, the history of the reported number of OPV doses received by each patient as reported by the caregiver might be inaccurate depending on caregiver recall and the history-taking methods of the investigator.

### Implications for Public Health Practice

Interruption of WPV1 transmission in Afghanistan will require implementation of high-quality house-to-house SIAs, with intense focus on identifying and vaccinating previously missed populations. The risk for WPV1 transmission in Afghanistan and Pakistan will continue as long as WPV1 circulation persists in either country; cross-border synchronization of surveillance and vaccination activities of both countries is essential to interrupting transmission in the two remaining countries with ongoing WPV1 transmission.

## Footnotes

* SIAs are mass immunization campaigns intended to supplement the routine immunization systems and generally target infants and children aged <5 years with OPV, regardless of their vaccination history. In countries with poliovirus circulation, children frequently receive multiple SIA doses per year. In Afghanistan, SIAs are conducted using a variety of modalities, such as house-to-house, mosque-to-mosque, and site-to-site vaccination.

† LQAS is a rapid survey method used to assess the quality of vaccination activities after SIAs in predefined areas (lots), such as health districts, using a sample size of 60. LQAS involves dividing the population into lots and ascertaining receipt of vaccination by randomly selecting children within each lot. If more than three children in the sample are unvaccinated, then the SIA quality in that area is classified as failed (i.e., at a pass threshold of ≥90%) and mop-up activities (i.e., search for children recently missed with a focal repeat house-to-house vaccination activity) are recommended. If the 90% threshold is met, the SIA’s quality for the area is classified as passed.

§ NPAFP cases are those that are discarded as not having laboratory or other proof of poliovirus as the cause. The expected background rate of NPAFP is two or more cases per 100,000 children aged <15 years per year, the standard WHO performance indicator target for sufficiently sensitive surveillance to detect a case of polio.

¶ Adequate stool specimens are defined as two stool specimens of sufficient quality for laboratory analysis, collected ≥24 hours apart, both within 14 days of paralysis onset (i.e., arriving in good condition at a WHO-accredited laboratory with reverse cold chain maintained, without leakage or desiccation, and with proper documentation). The global standard surveillance performance indicator target is ≥80% of AFP cases with adequate stool specimens collected.

** 45 C.F.R. part 46, 21 C.F.R. part 56; 42 U.S.C. Sect. 241(d); 5 U.S.C. Sect. 552a; 44 U.S.C. Sect. 3501 et seq.

††
https://polioeradication.org/news-post/global-eradication-of-wild-poliovirus-type-2-declared/

§§ Orphan viruses are ≥1.5% divergent from their closest genetic match (i.e., ≤98.5% identity) and can indicate gaps in AFP surveillance. The Global Polio Laboratory Network bases its findings on the analysis of the genetic divergence of the 906-nucleotide VP1 capsid protein coding region of poliovirus isolates.

## References

[1] Rachlin A, Patel JC, Burns CC, Progress toward polio eradication—worldwide, January 2020–April 2022. MMWR Morb Mortal Wkly Rep 2022;71:650–5. 10.15585/mmwr.mm7119a2 35552352 PMC9098249

[2] Global Polio Eradication Initiative. GPEI strategy 2022–2026. Geneva, Switzerland: World Health Organization; 2021. https://polioeradication.org/gpei-strategy-2022-2026/

[3] Mohamed A, Akbar IE, Chaudhury S, Progress toward poliomyelitis eradication—Afghanistan, January 2021–September 2022. MMWR Morb Mortal Wkly Rep 2022;71:1541–6. 10.15585/mmwr.mm7149a1 36480464 PMC9762895

[4] Sadigh KS, Akbar IE, Wadood MZ, Progress toward poliomyelitis eradication—Afghanistan, January 2020–November 2021. MMWR Morb Mortal Wkly Rep 2022;71:85–9. 10.15585/mmwr.mm7103a3 35051135 PMC8774155

[5] World Health Organization. Immunization Afghanistan 2023 country profile. Geneva, Switzerland: World Health Organization; 2023. https://www.who.int/publications/m/item/immunization-afghanistan-2023-country-profile

[6] Mbaeyi C, Baig S, Safdar RM, Progress toward poliomyelitis eradication—Pakistan, January 2022–June 2023. MMWR Morb Mortal Wkly Rep 2023;72:880–5. 10.15585/mmwr.mm7233a1 37590173 PMC10441828

[7] World Food Programme. WFP Afghanistan country brief. Geneva, Switzerland: World Food Programme; 2023. https://docs.wfp.org/api/documents/WFP-0000150932/download/

[8] Saleem AF, Mach O, Quadri F, Immunogenicity of poliovirus vaccines in chronically malnourished infants: a randomized controlled trial in Pakistan. Vaccine 2015;33:2757–63. 10.1016/j.vaccine.2015.04.055 25917673 PMC4447616

